# Ginsenoside Rg5 Inhibits Human Osteosarcoma Cell Proliferation and Induces Cell Apoptosis through PI3K/Akt/mTORC1-Related LC3 Autophagy Pathway

**DOI:** 10.1155/2021/5040326

**Published:** 2021-06-25

**Authors:** Ming-Yang Liu, Fei Liu, Yan-Jiao Li, Jia-Ning Yin, Yan-Li Gao, Xin-Yue Wang, Chen Yang, Jian-Guo Liu, Hai-Jun Li

**Affiliations:** ^1^Department of Immunity, Institute of Translational Medicine, The First Hospital of Jilin University, China; ^2^Departments of Orthopaedics, The First Hospital of Jilin University, China; ^3^Department of Obstetrics, The First Hospital of Jilin University, China; ^4^Department of Pharmacy, The First Hospital of Jilin University, China; ^5^Department of Pediatrics, The First Hospital of Jilin University, China; ^6^Department of Scientific Research, College of Basic Medicine, Jilin University, China

## Abstract

The function and mechanism underlying the suppression of human osteosarcoma cells by ginsenoside-Rg5 (Rg5) was investigated in the present study. MG-63, HOS, and U2OS cell proliferation was determined by MTT assay after Rg5 treatment for 24 h. Rg5 inhibited human osteosarcoma cell proliferation effectively in a dose-dependent manner. The range of effective inhibitory concentrations was 160-1280 nM. Annexin V-FITC and PI double-staining assay revealed that Rg5 induced human osteosarcoma cell apoptosis. Western blotting, qRT-PCR, and FACS experiments revealed that Rg5 inhibited human osteosarcoma cells via caspase-3 activity which was related to the LC3-mediated autophagy pathway. Rg5 decreased the phosphorylation of PI3K, Akt, and mTORC1 activation. In contrast, LC3-mediated autophagy and caspase-3 activity increased significantly. A PI3K/AKT stimulator, IGF-1, reversed Rg5-induced cell autophagy and apoptosis in MG-63 cells. Collectively, the current study demonstrated that Rg5 induced human osteosarcoma cell apoptosis through the LC3-mediated autophagy pathway. Under physiological conditions, activation of PI3K/AKT/mTORC1 inhibits LC3 activity and caspase-3-related cell apoptosis. However, Rg5 activated LC3 activity by inhibiting the activation of PI3K/AKT/mTORC1. The present study indicated that Rg5 could be a promising candidate as a chemotherapeutic agent against human osteosarcoma.

## 1. Introduction

Osteosarcoma (OS) causes a 2.4% death rate in child cancers, which is a fatal malignancy in pediatric patients [1]. The 5-year survival rate is no more than 70% because there are limited effective therapies except for surgical treatment [2]. OS is believed to be derived from malignant mesenchymal stem cells of the long bones [3–6]. Chemotherapy is still suitable for patients who are not suitable for surgery. However, side effects and drug resistance limited the use of chemotherapy drugs [7]. Herbal medicines and natural products have drawn increasing attention to novel anticancer agents because of the outstanding effectiveness and safety [8–10].

Ginseng has been used for over 2,000 years in various countries in East Asia, including Korea, China, Japan, and Vietnam [11]. Ginsenosides are the most effective components in ginseng. Rg5 is a minor ginsenoside but exhibits a superior pharmaceutical effect comparing with other major ginsenosides [12–14]. Rg5 promoted breast cancer cell apoptosis and inhibited cell proliferation by activating the AMPK pathway [15–17]. Through inducing G2/M phase cell cycle arrest and ROS-mediated MAPK activity, Rg5 inhibit human gastric cancer cell proliferation [18]. Rg5 also suppressed proliferation and promoted the apoptosis of human esophageal cancer cells and human hepatoma HepG2 cells [19, 20]. Additionally, inhibiting the AKT signaling pathway and downregulating BCL2 expression is the main mechanism of Rg5 on inhibition of retinoblastoma cells [21]. Therefore, Rg5 may serve as a chemosensitizer for reversing multidrug resistance [22].

Autophagy is considered to be an important cellular metabolic process [23, 24]. The function of autophagy regulates cell fate via different mechanisms [25, 26]. There is no study on the function of Rg5 on human osteosarcoma cells and the related mechanisms from now on. The current study investigated whether ginsenoside Rg5 induce the osteosarcoma cell apoptosis and the potential mechanisms. These findings may contribute to further understand the inhibitory effect of Rg5 against human osteosarcoma cell proliferation, as well as highlight the possibility of Rg5 as therapeutic agents for human osteosarcoma.

## 2. Material and Methods

### 2.1. Reagents

Rg5 was purchased from the Yuanye Pharmaceutical Company (#P186763-78-0, Shanghai, China). Rg5 was dissolved in dimethylsulfoxide (DMSO, #D8370, Solarbio, Beijing, China) at 100 mM and stored at -20°C. MTT (#M2003) and RNase (#R4875) were purchased from Sigma (Shanghai, China). Annexin V/PI kit was purchased from BD company (#556547, Franklin Lakes, NJ, USA). LC3 siRNA reagent kit (#6215), the primary and second antibodies used in western blotting, was purchased from CST (Boston, MA, USA). DMEM cell culture medium (#12491-023), FBS (#10099141), antibiotic for cell culture (#10378016), and trypsin (#25300054) were from GIBCO (Grand Island, NY, USA).

### 2.2. Cell Proliferation Assay

HOS, MG-63, and U2OS were purchased from ATCC in Shanghai of China. 5 × 10^4^ cells were seeded in a 96-well plate for 24 h, then treated with Rg5 (0, 10, 20, 40, 80, 160, 320, 640, and 1280 nM) for another 24 h for MTT assay. MTT (1.0 mg/mL) was added to each well, and the cells were incubated for 4 h. The MTT solution was then aspirated, and 100 *μ*L DMSO was then added. The 96-well plates were read using a microplate spectrophotometer (Synergy H1, BioTek, USA) at 540 nm. The inhibition percentage was calculated as (1 − the value in experimental group/the value in the control group) × 100%.

### 2.3. FCM Experiment

Annexin V-FITC and PI double staining flow cytometry analyses were employed to assess cell apoptosis. MG-63, HOS, and U2OS cells were collected on the experimental endpoint. Cell apoptosis was analyzed using a flow cytometer (FACScan; BD Biosciences, Franklin Lakes, NJ, USA) with FlowJo 7.6 FACS analysis software (FlowJo LLC, Ashland, OR, USA). For LC3 staining, cells were stained with FITC-conjugated mouse anti-human LC3 (#bs-8878R, BIOSS). All antibodies were 100-fold dilutions and incubated in 4°C for 15 mins.

### 2.4. Western Blotting

Cell total protein was extracted, and the protein concentration was determined according to the manufacturer's protocol. A total of 5-40 *μ*g cell total protein was separated by 10% SDS-PAGE and then electrophoretically transferred to polyvinylidene fluoride membranes (0.45 *μ*m; EMD Millipore, Billerica, MA, USA) and blocked at 37°C for 1 h with 5% skim milk in Tris-buffered saline (TBS) with Tween-20 (0.1%). Caspase-3 (#9662), cleaved caspase-3 (#9661), LC3 (#4108), PI3K (#4225), p-PI3K (#13857), AKT (#4691), p-AKT (#4060), mTOR (#2972), Raptor (#2280), and *β*-actin (^#^4970) expression levels were determined semiquantitatively by densitometric analysis with the Quantity One software (V4.62, Bio-Rad Laboratories, Inc., Hercules, CA, USA).

### 2.5. qPCR Experiment

Total cellular RNA was isolated by TRIzol (Invitrogen, Carlsbad, USA). Total RNA was transcribed to cDNA by a reverse transcription kit (TransGen, Beijing, China). qRT-PCR was conducted using the Fast Start Universal SYBR Green Master (ROX) kit (Roche, Shanghai, China). Reactions were performed using 3 *μ*L of cDNA in a 20 *μ*L reaction volume and the following thermal cycle profile: 10 seconds for predenaturation at 94°C, 5 seconds for denaturation at 94°C, and 30 seconds for extension at 60°C, for 40 cycles. The primer sequences are shown as follows: LC3 sense, 5′-GACCATCTGGTTCAGGTTCC-3′, and antisense, 5′-ACATTCCCG AAACTCAGTCG-3′; caspase-3 sense, 5′-CCTCTGACTTCCAGGTGGTCT-3′, and antisense, 5′-TTCGTTGTGTTGCTGTAGCCAAA-3′; and GAPDH sense, 5′-CCAGGTGGTCTCCTCTGACTT-3′, and antisense, 5′-GTTGCTGTAGCCAAATTCGTTGT-3′. All primers were synthesized by Shanghai Sangon in China. The length of caspase-3 is 159 bp, and the PCR products were analyzed by agarose gel electrophoresis.

### 2.6. RNA Interference Experiment

The sequence of LC3-specific small interfering RNA (siRNA) reagent and the scramble control (GenePharma, Shanghai, China) is as follows: LC3 siRNA sense, 5′-GUGCAUCAGAUUUCATT-3′, and antisense, 5′-UGAAAAGC UCUGCACTT-3′; scramble sense, 5′-GCUCAUCATTGUGCAGA-3′, and antisense, 5′-UGAGCCACTUGAAAUCT-3. The MG-63 cell line was planted in a 12-well plate at a 5 × 10^4^/mL density and was transfected with 50 pmol of LC3 siRNA or scramble control using Lipofectamine 2000 (Life Technologies, Gaithersburg, MD, USA) and then incubated for 6 h. LC3 expressions were detected using a western blotting experiment.

### 2.7. Statistical Analysis

All data are shown as Mean ± Standard Deviation (M ± SD) and analyzed using the D'Agostino and Pearson omnibus normality test with at least three independent repetitions. Mean values were compared using either a paired *t*-test (two groups) or ANOVA (more than two groups). *p* < 0.05 were considered to be significant.

## 3. Results

### 3.1. Rg5 Inhibited Cell Growth and Induced Human Osteosarcoma Cell Apoptosis

**Figure 1:**
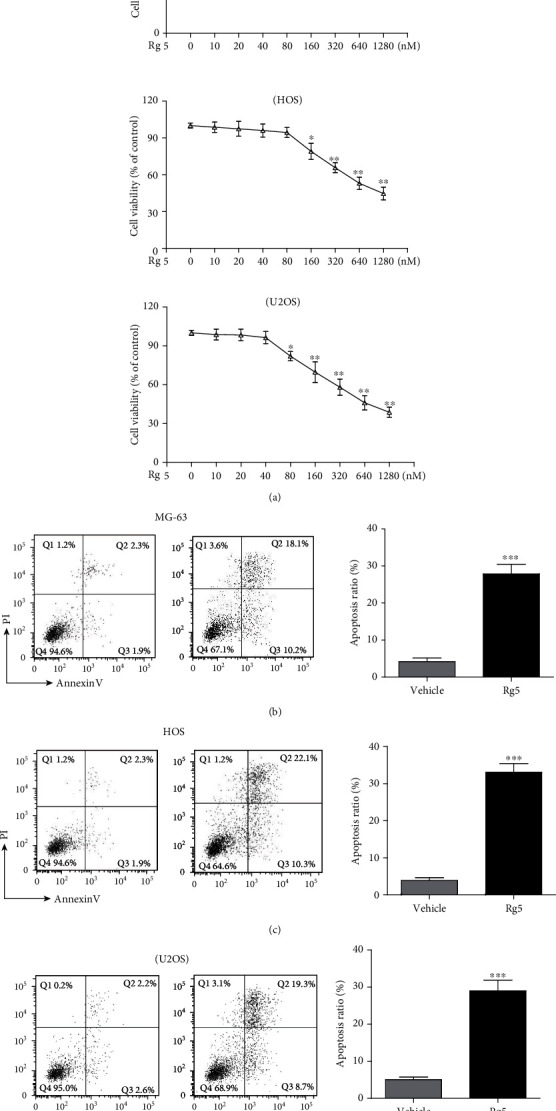
Rg5 inhibited human osteosarcoma cell growth and induced apoptosis. (a) MG-63 cells, HOS cells, and U2OS cell vitality were tested by MTT after 24 h treatment of Rg5 with various doses. The concentration for flow cytometric experiment is 160 nM. Representative figures and statistical analysis of the percentage of apoptotic cells in MG-63 (b), HOS (c), and U2OS (d) cells. The data are shown as M ± SD (*n* = 3, ^∗^*p* < 0.05, ^∗∗^*p* < 0.01, and ^∗∗∗^*p* < 0.001 vs. control).

MG-63, HOS, and U2OS cells were treated with Rg5 (0, 10, 20, 40, 80, 160, 320, 640, and 1280 nM) for 24 h; cell proliferation was determined by MTT assay (Figure 1(a)). Rg5 significantly inhibited the growth of MG-63 cells at doses of 80-1280 nM (*p* < 0.01). The susceptibility of HOS cells to Rg5 was slightly weaker than MG-63 cells. The range of effective inhibitory concentrations was 160-1280 nM (*p* < 0.01). The susceptibility of U2OS is similar with MG-63 cells, and the range of effective inhibitory concentrations was 80-1280 nM (*p* < 0.001). Based on the MTT results, we chose 160 nM of Rg5 as the working concentration for apoptosis detection after 24 h treatment. The numbers of apoptotic cells ranged from 4.5 ± 0.8% to 29.9 ± 3.1% in MG-63 cells (Figure 1(b), *p* < 0.001), 3.4 ± 0.9% to 32.3 ± 3.9% in HOS cells (Figure 1(c), *p* < 0.001), and 4.6 ± 1.1% to 27.2 ± 3.5% in U2OS cells (Figure 1(d), *p* < 0.001).

### 3.2. Rg5 Inhibited Human Osteosarcoma Cells via Caspase-3 Activity Related to the LC3 Autophagy Pathway

**Figure 2:**
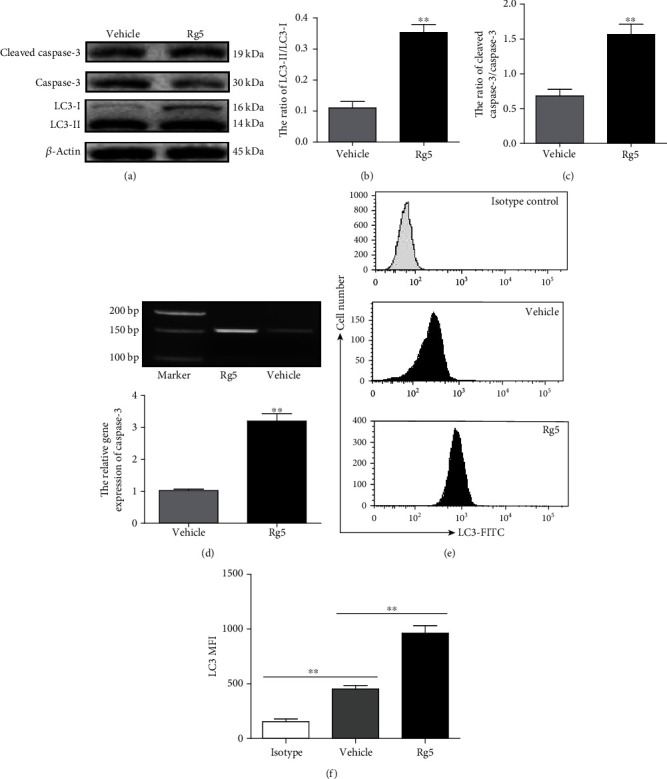
Rg5 inhibited MG-63 cells via caspase-3 activity that was related to the LC3-mediated autophagy pathway. MG-63 cells were treated with 160 nM of Rg5, and total proteins were extracted. The expression levels related proteins were detected by western blotting, qRT-PCR, or FACS. (a) Representative image of proteins detected by western blotting. The ratio of LC3-II/LC3-I (b) and cleaved caspase-3/caspase-3 (c) in MG-63 cell. (d) The relative gene expression of caspase-3. (e, f) The MFI of LC3 detected by FACS. The data are shown as M ± SD (*n* = 3, ^∗∗^*p* < 0.01 vs. control).

MG-63 cells, as a representative cell of osteosarcoma, were chose for the mechanism study. MG-63 cells were treated with Rg5 (160 nM) for 12 h. Caspase-3 and LC3 autophagy-related protein was detected by western blotting, qRT-PCR, or FACS. The ratio of LC3-II/LC3-I increased approximately 3-fold after Rg5 treatment (Figures 2(a) and 2(b), *p* < 0.01), accompanied by cleaved caspase-3 activity (Figures 2(a) and 2(c), *p* < 0.01). Caspase-3 gene expression was also confirmed by qRT-PCR experiments (Figure 2(d), *p* < 0.01). Fluorescence-labeled flow cytometry for LC3 expression showed that Rg5 significantly increased the mean fluorescence intensity (MFI) of LC3 in MG-63 cells (Figures 2(e) and 2(f), 960 ± 50 vs. 450 ± 30, *p* < 0.01).

### 3.3. Inhibition of Autophagy Reduced Rg5-Induced Caspase-3 Activity and Cell Apoptosis

**Figure 3:**
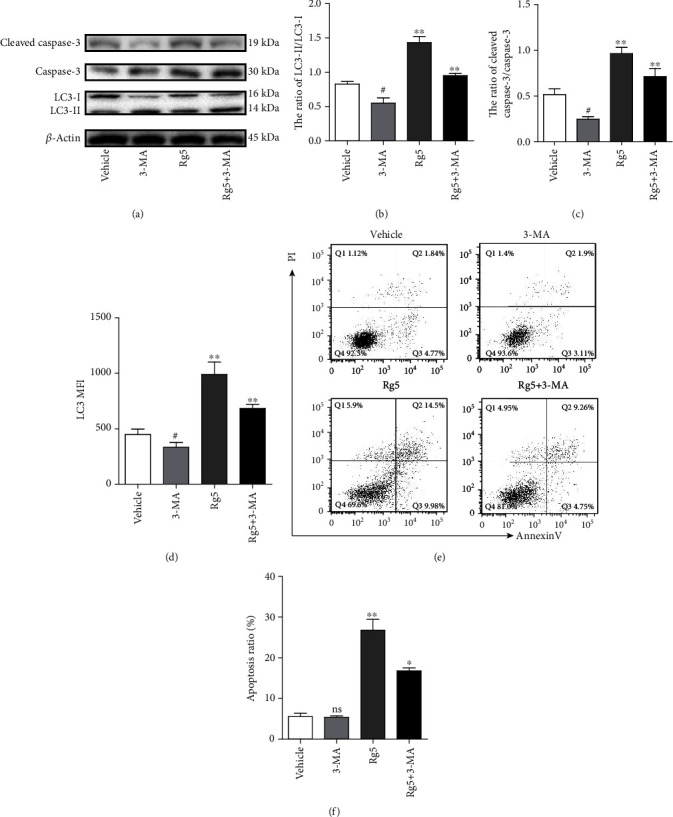
Inhibition of autophagy reduced Rg5-induced caspase-3 activity and cell apoptosis. 5 mM 3-MA combining or alone with 160 nM Rg5 was used to treat MG-63 cells for 12 h and for caspase-3 and LC3 autophagy-related protein detection by western blotting or FACS. (a) Representative image of proteins detected by western blotting. (b) The ratio of LC3-II/LC3-I in MG-63 cells. (c) The ratio of cleaved caspase-3/caspase-3 in MG-63 cells. (d) The MFI of LC3 in MG-63 cells detected by FACS. (e, f) MG-63 cell apoptosis detected by FACS. The data are shown as M ± SD (*n* = 3, ^ns^*p* > 0.05, ^#^*p* < 0.05, 3-MA treatment comparing with control; ^∗^*p* < 0.05, ^∗∗^*p* < 0.01 vs. control).

3-MA acts as an inhibitor of autophagy. 5 mM 3-MA alone or combining with 160 nM Rg5 was used to treat MG-63 cells for 12 h and for caspase-3 and LC3 autophagy-related protein detection by western blotting, qRT-PCR, or FACS. The ratio of LC3-II and LC3-I was reduced when 3-MA was added to the Rg5 treatment system (Figures 3(a) and 3(b), *p* < 0.01). The inhibition effect of 3-MA on LC3-mediated autophagy was also confirmed by fluorescence-labeled flow cytometry (Figures 3(e) and 3(f), *p* < 0.01). Interestingly, when LC3 activity was blocked by 3-MA, caspase-3 activity was also reduced at the protein level (Figure 3(c), *p* < 0.01) compared with Rg5 alone. Furthermore, cell apoptosis staining assays showed that 3-MA decreased Rg5-induced MG-63 cell apoptosis (Figures 3(e) and 3(f), *p* < 0.01).

### 3.4. Silencing LC3 Affects Rg5-Induced Tumor Cell Apoptosis

**Figure 4:**
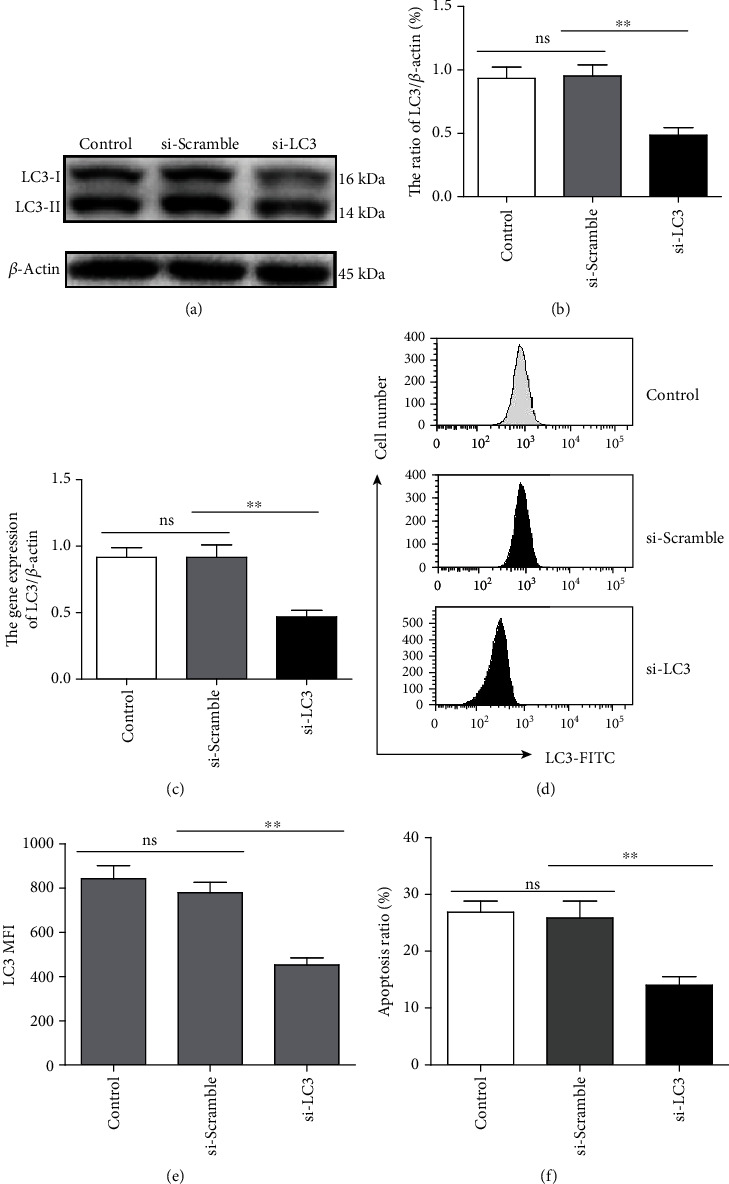
Silencing LC3 affects Rg5-induced tumor cell apoptosis. LC3 protein and gene expression were detected by western blot (a, b) and qRT-PCR experiments (c) when the LC3 expression was silenced by RNAi in MG-63 cells. (d, e) The MFI of LC3 in MG-63 cells detected by FACS. (f) MG-63 cell apoptosis detected by FACS. The data are shown as M ± SD (*n* = 3, ^ns^*p* > 0.05, ^∗∗^*p* < 0.01 vs. control).

LC3 protein and gene expression were decreased significantly by western blot (Figures 4(a) and 4(b), *p* < 0.01) and qRT-PCR experiments (Figure 4(c), *p* < 0.01) when the LC3 expression was silenced by RNAi in MG-63 cells. The MFI level of LC3 also decreased significantly (Figures 4(d) and 4(e), *p* < 0.01). The numbers of apoptotic cells decreased significantly in LC3-silenced MG-63 cells compared to those of the control cells (Figure 4(f), *p* < 0.01). These results proved that the LC3 autophagy pathway is a key factor in MG-63 cell apoptosis initiated by Rg5.

### 3.5. The PI3K/AKT/mTORC1 Pathway Is Involved in Rg5-Induced Human Osteosarcoma Cell Autophagy Activation and Apoptosis

**Figure 5:**
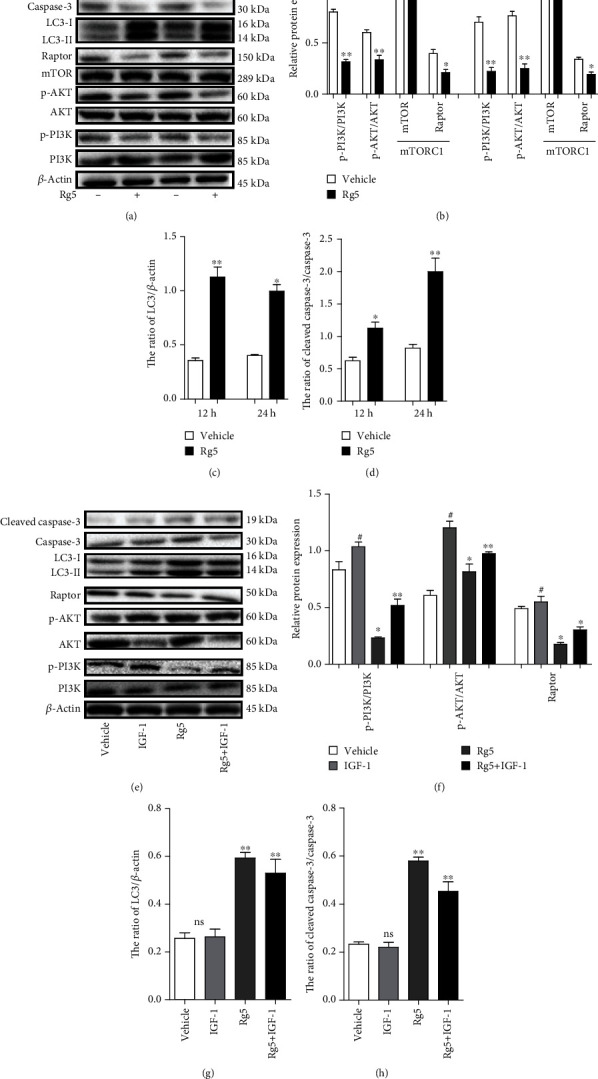
The PI3K/AKT/mTORC1 pathway is involved in Rg5-induced human osteosarcoma cell autophagy activation. (a) Representative image of proteins detected by western blotting at 12 h and 24 h after the treatment. (b) Phosphorylated PI3K, Akt, and TORC1 in MG-63 cells. (c) The ratio of LC3/*β*-actin in MG-63 cells. (d) The ratio of cleaved caspase-3/caspase-3 in MG-63 cells. (e, f) Phosphorylated PI3K, Akt, and TORC1 in MG-63 treated with IGF-1 were detected by WB at 24 h after the treatment. (g) The ratio of LC3/*β*-actin in MG-63 cells. (h) The ratio of cleaved caspase-3/caspase-3 in MG-63 cells. The data are shown as M ± SD (*n* = 3, ^#^*p* < 0.05 IGF treatment comparing with control; ^ns^*p* > 0.05, ^∗^*p* < 0.05, ^∗∗^*p* < 0.01 vs. control).

**Figure 6:**
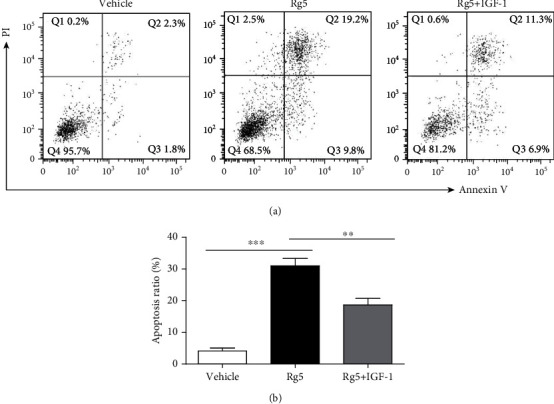
The PI3K/AKT/mTORC1 pathway is involved in Rg5-induced human MG-63 cell apoptosis. (a, b) Cell apoptosis of MG-63 cells treated with Rg5 alone or together with IGF-1 was examined by FACS. The data are shown as M ± SD (*n* = 3, ^ns^*p* > 0.05, IGF treatment comparing with control, ^∗∗^*p* < 0.01, ^∗∗∗^*p* < 0.001 vs. control).

mTORC1 is a complex consisting of four proteins, including mTOR, Raptor, G*β*L, and DEPTOR [27]. Downregulation of phosphorylated PI3K (*p* < 0.05) and phosphorylated Akt (*p* < 0.05) and upexpression of Raptor (*p* < 0.01), meaning of mTORC1 activation, unless the stable expression of mTOR, were observed by western blot assay on MG-63 cell treatment with Rg5 for 12 h and 24 h in Figures 5(a) and 5(b). In contrast, LC3-mediated autophagy (Figures 5(a) and 5(c), *p* < 0.01) and caspase-3 activity (Figures 5(a) and 5(d), *p* < 0.01) increased significantly when MG-63 cells were treated with Rg5. To further confirm that Rg5 induced LC3-mediated autophagy and caspase-3 activity by inhibiting PI3K/AKT/mTOR phosphorylation, we used the PI3K activator IGF-1 combined with Rg5 treatment in MG-63 cells. Not surprisingly, IGF-1 reversed the Rg5-induced reduction in PI3K, Akt, and Raptor activation (Figures 5(e) and 5(f), *p* < 0.01). Moreover, the LC3 and caspase-3 activity decreased when IGF-1 was added compared with that of Rg5 treatment alone (Figures 5(g) and 5(h), *p* < 0.01). Cell apoptosis was evaluated by Annexin V-FITC and PI double-staining. As shown in Figures 6(a) and 6(b), the numbers of apoptotic cells decreased significantly when IGF-1 was used together with Rg5 (*p* < 0.01). These results indicated that Rg5 inhibited the PI3K/AKT/mTORC1 pathway and then induced human osteosarcoma cell autophagy activation and cell apoptosis.

## 4. Discussion and Conclusion

Osteosarcoma (OS) causes a 2.4% death rate in child cancers worldwide. At present, there remain a number of side effects of chemotherapeutic drugs in human osteosarcoma treatment [28]. Natural medicines have some advantage in the cancer treatment to overcome the associated side effects [8]. So the increasing interest has drawn more and more attention for novel anticancer agents from natural products [9].

The present study investigated the antiproliferative and apoptosis-inducing effects of ginsenoside Rg5 on human osteosarcoma cells. MG-63, HOS, and U2OS were chose as target cells. Rg5 inhibited MG-63, HOS, and U2OS cell proliferation at concentrations ranging from 80 to 1280 nM. The susceptibility of HOS cells to Rg5 was slightly weaker than that of MG-63 and U2OS cells. Cell apoptosis was closely related to cell proliferation inhibition [29, 30]. FACS experiment revealed that Rg5 significantly induced human osteosarcoma cell apoptosis. To observe the mechanism of Rg5 on osteosarcoma cell apoptosis, the caspase-3 gene was detected. As expected, Rg5 significantly increased the activation of caspase-3. Autophagy is considered to be an important cellular metabolic process [24]. The function of autophagy can regulate cell fate via different mechanisms [25, 31]. The present study revealed that the ratio of LC3-II/LC3-I increased approximately 3-fold after Rg5 treatment, accompanied by cleaved caspase-3 activity. These results suggest that autophagy may be associated with caspase-3-related cell apoptosis. 3-MA, an LC3-mediated autophagy inhibitor, was used to treat MG-63 cells combining with Rg5. As expected, cleaved caspase-3 activity and cell apoptosis were significantly reduced when LC3-mediated autophagy was inhibited by 3-MA. This mechanism was also confirmed in LC3 RNA-silenced MG-63 cells.

More and more novel anticancer agents from natural products have been used in osteosarcoma treatment. Sapio et al. demonstrate that CGA acts as an anticancer molecule affecting the cell cycle and provoking cell growth inhibition mainly by apoptosis induction by ERK1/2 activation [32]. They also reported AdipoR affected osteosarcoma cell cycle and cell death in the mTORC1 pathway [33]. Akt signaling pathways are responsible for antiproliferative actions in some cells [34]. Although there is no study on Rg5 on the Akt signaling pathway, a previous study revealed that Rg3 attenuates lipopolysaccharide-induced acute lung injury via activation of the PI3K/AKT/mTOR pathway [35]. To further verify which signaling molecules are related to MG-63 cell autophagy and apoptosis, the current study examined the expression of MAPK components and AKT using western blotting. Downregulation of phosphorylated PI3K, phosphorylated Akt, and phosphorylated TORC1 was observed using western blotting after 12 h and 24 h treatment of MG-63 cells with Rg5. In contrast, LC3 autophagy and caspase-3 activity increased significantly. To further confirm that Rg5 induced LC3-mediated autophagy and caspase-3 activity by inhibiting PI3K/AKT/mTOR phosphorylation, we used the PI3K activator IGF-1 combined with Rg5 treatment in MG-63 cells. LC3 and caspase-3 activity decreased when IGF-1 was added compared with that of Rg5 treatment alone. Cell apoptosis, cell migration ability, and invasion ability decreased significantly when IGF-1 was used together with Rg5. These results revealed that Rg5 inhibited the PI3K/AKT/mTORC1 pathway, induced human osteosarcoma cell autophagy activation, and induced cell apoptosis.

**Figure 7:**
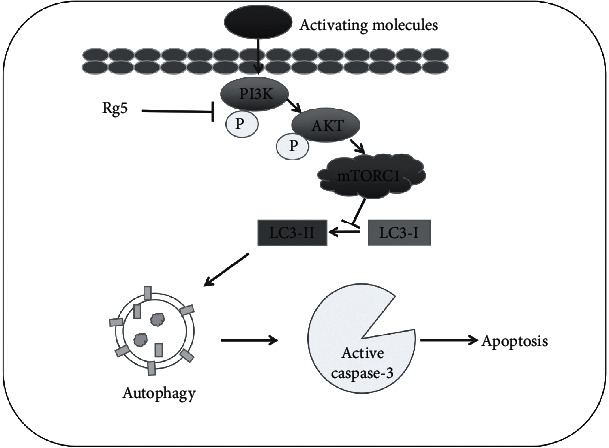
Schematic outline of ginsenoside Rg5 inhibits the human osteosarcoma cell through LC3 autophagy-related apoptosis. Under physiological conditions, the activation of PI3K/AKT/mTORC1 could inhibit the LC3 activity and caspase-3-related cell apoptosis. But Rg5 activated LC3 activity through inhibiting the phosphorylation of PI3K/AKT/mTORC1 to inhibit the human osteosarcoma cell proliferation and induce cell apoptosis.

In conclusion, these data demonstrated that Rg5 inhibited proliferation and induced apoptosis through the LC3 autophagy pathway. Under physiological conditions, the activation of PI3K/AKT/mTORC1 could inhibit the LC3 activity and caspase-3-related cell apoptosis. But Rg5 activated LC3 activity through inhibiting the phosphorylation of PI3K/AKT/mTORC1 (Figure 7). The present study indicated that Rg5 could be a promising candidate as a chemotherapeutic agent against human osteosarcoma.

## Data Availability

The data used to support the findings of this study are available from the corresponding author upon request (Dr. Haijun Li, hjli2012@jlu.edu.cn).

## Abbreviations

Rg5: Ginsenoside-Rg5
MTT: Methylthiazolyldiphenyl-tetrazolium bromide
qRT-PCR: Real-time quantitative PCR
FACS: Fluorescence activated cell sorter
OS: Osteosarcoma
ROS: Reactive oxygen species
FBS: Fetal bovine serum
PI: Propidium iodide
MFI: Mean fluorescence intensity.
